# Examining Bloom’s Taxonomy in Multiple Choice Questions: Students’ Approach to Questions

**DOI:** 10.1007/s40670-021-01305-y

**Published:** 2021-05-25

**Authors:** J. K. Stringer, Sally A. Santen, Eun Lee, Meagan Rawls, Jean Bailey, Alicia Richards, Robert A. Perera, Diane Biskobing

**Affiliations:** 1 Office of Assessment, Evaluation, and Scholarship, Virginia Commonwealth University School of Medicine, Richmond, VA USA; 2 Office of Integrated Medical Education, Rush Medical College, Chicago, IL USA; 3 Department of Immunology, Virginia Commonwealth University School of Medicine, Richmond, VA USA; 4 Office of Faculty Development, Virginia Commonwealth University School of Medicine, Richmond, VA USA; 5 Department of Biostatistics, Virginia Commonwealth University School of Medicine, Richmond, VA USA; 6 Department of Internal Medicine, Division of Endocrinology, Virginia Commonwealth University School of Medicine, Richmond, VA USA

**Keywords:** Medical students, Multiple choice questions, Clinical reasoning, Assessment, Bloom's taxonomy

## Abstract

**Background:**

Analytic thinking skills are important to the development of physicians. Therefore, educators and licensing boards utilize multiple-choice questions (MCQs) to assess these knowledge and skills. MCQs are written under two assumptions: that they can be written as higher or lower order according to Bloom’s taxonomy, and students will perceive questions to be the same taxonomical level as intended. This study seeks to understand the students’ approach to questions by analyzing differences in students’ perception of the Bloom’s level of MCQs in relation to their knowledge and confidence.

**Methods:**

A total of 137 students responded to practice endocrine MCQs. Participants indicated the answer to the question, their interpretation of it as higher or lower order, and the degree of confidence in their response to the question.

**Results:**

Although there was no significant association between students’ average performance on the content and their question classification (higher or lower), individual students who were less confident in their answer were more than five times as likely (OR = 5.49) to identify a question as higher order than their more confident peers. Students who responded incorrectly to the MCQ were 4 times as likely to identify a question as higher order than their peers who responded correctly.

**Conclusions:**

The results suggest that higher performing, more confident students rely on identifying patterns (even if the question was intended to be higher order). In contrast, less confident students engage in higher-order, analytic thinking even if the question is intended to be lower order. Better understanding of the processes through which students interpret MCQs will help us to better understand the development of clinical reasoning skills.

## Introduction

Students may enter medical school equipped with a range of test-taking strategies developed over years of taking multiple choice questions (MCQs) in various settings. They may utilize a variety of approaches such as pattern recognition, buzzword identification, and rote memorization. While memorization of some topics such as anatomy and microbiology is necessary during medical school and in practice, it is not sufficient for the practice of medicine, much less to succeed on most medical school and licensing exams [1]. These exams typically require a different approach to test taking that necessitates the use of higher-order thinking skills to apply their underlying knowledge. Development of higher-order thinking skills is related to student academic success [2]. Therefore, many medical school faculty strive to write MCQs that entail the application of analytic thinking skills in an effort to model skills needed for clinical reasoning [3, 4].

As a group of medical educators who attempt to write high-level questions in this manner, we believed that lower-performing students would be more likely to perceive questions as higher order. However, given that students may perceive items differently than what we intend when we write them, we began this study as a way to understand student perspectives and to identify students who may need more support transitioning into the kinds of higher-order thinking expected of physicians.

Bloom’s taxonomy is frequently used to describe educational objectives [5, 6] based on a hierarchy of thinking skills (remember, understand, apply, analyze, evaluate, create) that has become a framework for writing questions in education. Faculty may attempt to write questions to target a specific level of the taxonomy [7], but writing questions that target higher levels of the taxonomy can be challenging [8–10]. For example, faculty in one study approached MCQ writing at lower- and higher-order thinking levels with the intention of testing clinical reasoning skills and information recall, but rewriting was necessary to achieve a balance between question difficulty and lower- or higher-order classification [11, 12].

The National Board of Medical Examiners Item Writing Guide ultimately classifies questions based on “application of knowledge vs recall of a fact” rather than identifying a specific taxonomic level [13]. “The selection of item types depends on the intent of their use: for a medium- to high-stakes summative examination, the use of vignettes that require higher-order thinking skills and application of knowledge would be preferable to simple recall items.” [13 p. 32] MCQs requiring application of knowledge are utilized because they are thought to be a reliable measure of clinical reasoning [3]. Learners need to have basic knowledge (facts) in order to approach higher-order questions; in other words, they have to walk before they can run. Lower-order facts are necessary but not sufficient for higher-order reasoning. By the time students reach residency, most should have developed clinical reasoning skills necessary to help them critically evaluate, diagnose, and treat patients. Resident physicians have been found to be adept at distinguishing between higher- and lower-order MCQs and apply clinical reasoning skills or recall knowledge appropriately [14]. This implies that at some point in their learning, residents transition from walking to running, but where does that shift happen? We must also question if the dichotomization of Bloom’s taxonomy into higher or lower order is the appropriate tool to observe these kinds of changes. Given that one’s knowledge in an area may or may not actually allow them to distinguish between higher- or lower-order questions, what factors can we use to monitor this transition?

As every learner is different in their understanding, knowledge, and approach to learning, they may perceive questions differently, especially on the road from first-year medical student to resident and beyond. We believe that all learners will eventually make the transition from lower- to higher-order thinking as a byproduct of the complex and dynamic clinical learning environments. What is less certain is when and how educators can support this development in the undergraduate medical education context. Understanding this development will help us to deepen the teaching of clinical reasoning for advanced learners and provide additional support to learners who might be struggling.

There is a commonly held belief that faculty can write a question that targets a specific learning level, Bloom’s higher- or lower-order, but this belief assumes that students interpret items the same way that faculty intend without taking learner characteristics, such as performance or development, into account. One study examined how interns employed clinical reasoning strategies and test-taking behaviors while solving clinical vignette MCQs by analyzing “thinking aloud” comments during MCQ problem-solving sessions [15]. They noted that high performers rule out alternative answers whereas low performers select response options too quickly. Zaidi et al. [16] argue that despite the intended level of question, students often employ higher-level thinking skills to answer both lower- and higher-order questions. In this study, we aimed to explore first-year medical students’ perceptions of MCQs to provide a baseline for understanding the development of analytic thinking from undergraduate medical education into practice. This knowledge may help faculty develop questions that better facilitate analytic thinking.

To understand the full scope of student performance on MCQ exams, it is important to explore MCQs from a lens of student perceptions and performance. We used Bloom’s taxonomy to classify MCQs as lower order (remember, understand) or higher order (apply, analyze, evaluate, create) [5, 6]. Given the framework established by Zaidi et al. [16], we analyzed students’ perceptions of MCQs and the way those perceptions might be related to performance. To further explore this area, the study team developed three questions:

1. Do first year medical students who get the questions incorrect approach the question in the same manner as the instructor intended?
2. Do first year medical students who are not confident in the answer or who do not get the answer correct approach question as higher order?
3. Do first year medical students with less knowledge (lower score on total exam) approach more questions as higher order?

## Methods

Twenty practice questions for an upcoming exam in the endocrine block were developed by a course instructor in an effort to write MCQs of both high and low difficulty that were intended to test higher- and lower-order thinking. Following the creation of these MCQs, all 20 were reviewed by three clinicians and three educators, with variable expertise in endocrinology, until consensus about classification as higher or lower order was reached. There were 10 higher and 10 lower order. This consensus building was an important step in the process. As Tractenberg et al. suggest [11], expertise in content knowledge alone may leave experts ill-equipped to identify MCQs as higher or lower order. We believe that by collaboratively assigning labels to items, we can improve the validity of inferences drawn about these classifications.

An optional course review session open to the entire class was offered prior to an upcoming exam. Following this review session, first-year medical students at the review who volunteered to participate were provided with a brief overview and handout describing Bloom’s taxonomy, as well as examples of lower-order and higher-order questions to help them understand the levels of learning in Bloom’s taxonomy. The sample consisted of 137 first-year medical students (74% of the class participated). Students were informed that participation was voluntary but were offered information on their performance if they completed the survey.

Students were then given the 20-item practice exam. Students first answered a question, then, immediately following their answer they were asked to identify whether they believed the item to be higher or lower order and to rate their confidence in their answer to the question using a 5-point Likert scale ranging from not confident at all to extremely confident. The process was then repeated with each question. Upon completion of the practice exam, the correct responses were provided to the group of students to review and discuss the items. In addition, approximately 2 weeks later, students were provided individual feedback about their performance on the practice items as well as the concordance between their rating of Bloom’s taxonomy and the faculty rating. They were also provided data on their performance on higher- and lower-order questions as defined by the faculty.

To examine whether students who answer questions incorrectly perceive the question in the same manner as the instructor intended (research question 1), a binomial generalized linear mixed model was used. This model was chosen for its ability to include a random effect of student that accounts for responses to questions being nested within students. The analysis was stratified using Bloom’s level (high vs low as assigned by the research team) to account for potential differences in the association depending on Bloom’s level. A stratified analysis allows for differing effects by question type while also allowing for the results to be easy to interpret. Similarly, in order to examine whether students who report low confidence in their answer or give an incorrect response to a question perceived questions as higher order (research question 2), a binomial generalized linear mixed model was used. Both variables were included in the model to account for any shared effect the variables may have with confidence being treated as a categorical variable. A stratified analysis was not conducted for this research question because two conditions (confidence and correctness) were included in identifying their approach to question as higher order. For each generalized linear mixed model, the odds ratios, 95% confidence intervals, and *p* values were reported. Lastly, a simple regression was performed to assess whether students’ knowledge (total exam score) was associated with the number of questions they perceived as higher order (research question 3). All tests were performed at *α* = 0.05 significance level and all analyses were performed using the R statistical software. The study was IRB exempt by Blinded Institution IRB (HM20015714).

## Results

The mean performance on the practice exam was 74.9% correct (SD = 10%). Average agreement with faculty Bloom’s level assignment was 70.2% (SD = 9%). These results demonstrate differences in student perceptions of the questions compared to faculty rating. This disparity suggested that students’ ability to answer a question correctly influenced their rating of the question as higher or lower order. For example, because so many students were readily able to answer question 8, they may have been more inclined to think it was lower order. Our hypothesis was that while the question was intended to be higher order, students likely used pattern recognition techniques to answer the question rather than analytic thinking. Binomial generalized linear mixed modeling was used to further test these relationships. Results for each model are provided in Tables 1, 2, and 3.

**Table 1:** Responses for how students getting questions incorrect approach the question

Question	Model	Outcome	Predictor	Odds Ratio	Confidence Interval	P-value
1: Do first year medical students who get the questions incorrect, approach the question in the same manner as the instructor intended?			Student Answered Correctly			
	1	Instructor’s Blooms Response Lower Ordered Questions Only	No	0.39	(0.28, 0.56)	<.0001
			Yes	Reference	Reference	
			Student Answered Correctly			
	2	Instructor’s Blooms Response Higher Ordered Questions Only	No	3.8	(2.9, 5.0)	<.0001
			Yes	Reference	Reference	

Interpretation of odds ratio values is based on a reference group such that groups with an odds ratio greater than one are more likely to indicate a response while groups with an odds ratio less than one are less likely to indicate a response than the reference group

**Table 2:** Relationship between student confidence and faculty Bloom’s alignment

Question	Model	Outcome	Predictor	Odds Ratio	Confidence Interval	P-value
2: Do first year medical students who are not confident in the answer or who do not get the answer correct approach question as higher order?	3	Student’s Blooms Response-Perceiving Question as Higher Order	Student’s Confidence level			
			Low-1	7.8	(5.50,11.04)	<.0001
			2	^a^	^a^	^a^
			3	2.55	(2.01,3.23)	<.0001
			4	5.49	(4.22,7.15)	<.0001
			High-5	Reference	Reference	
			Student Answered Correctly			
			No	2.95	(2.39,3.63)	<.0001
			Yes	Reference	Reference	

Interpretation of odds ratio values is based on a reference group such that groups with an odds ratio greater than one are more likely to indicate a response while groups with an odds ratio less than one are less likely to indicate a response than the reference group

^a^No estimate as response choice not observed

**Table 3:** Relationship

Question	Model	Outcome	Predictor	Beta	Standard error	*P* value
3: Do first year medical students with less knowledge (lower score on total exam) approach more questions as higher order?	4	Sum of higher-order questions	Number of questions incorrect	−0.13	0.12	0.3122

- Research question 1: Do first year medical students who get the questions incorrect approach the question in the same manner as the instructor intended?

◦For lower ordered questions, students who answered a question incorrectly had lower odds (OR = 0.39) of identifying the question as a low-order question. For higher-order questions, students with incorrect answers had four times as high odds of (OR = 3.8) perceiving the question as intended (Table 1). These results indicate that students who answer incorrectly tend to report the question as a higher-order question regardless of the intended question type.

- Research question 2: Do first year medical students who are not confident in the answer or who do not get the answer correct approach the question as higher order?

◦For the multivariable model, when regressing the students’ perceptions of the question on confidence with their answers and whether they answered correctly, students who were not confident at all (those students reporting a confidence level of one out of five) had 5.49 as high odds (OR = 5.49) of perceiving a question as higher order than those students who were extremely confident. Students who were less confident (confidence level three or four out of five) were also more likely to perceive a question as higher order compared to those who responded as extremely confident (students reporting a confidence level of five). Additionally, students who answer incorrectly have about three times the odds (OR = 2.95) of perceiving the question as higher order compared to students who answer correctly (Table 2).

- Research question 3: Do first year medical students with less knowledge (lower score on total exam) approach more questions as higher order?

◦No statistically significant association was found between the total knowledge (exam score) and the number of questions perceived as higher order (Table 3). Students who scored lower on content knowledge did not identify more questions as higher order compared to students who performed better.

## Discussion

Utilizing Bloom’s taxonomy to develop MCQs has been promoted as a mechanism to test higher order thinking skills. In this study, we evaluate whether students can identify MCQs as requiring higher- or lower-order thinking and the relationship to student performance. Faculty intentionally wrote higher- or lower-order questions as the gold standard. We demonstrated that students who are not confident in their answers, or who answer incorrectly, are more likely to perceive the question as higher order, whereas better performing and more confident students are more likely to perceive questions as lower -order. Importantly, our results found that students with lower levels of question performance were more likely to identify items as higher order than their more knowledgeable peers. This finding is consistent with Zaidi et al.’s [16] proposal that the difference in applying Bloom’s taxonomy between faculty and students is related to depth and breadth of foundational knowledge. Given their broader knowledge base, faculty seem to use different criteria for classifying items than students do. These findings suggest that faculty should ensure they take into account students’ depth of knowledge when writing questions. Our study also demonstrated that if a student gets a question correct, they are less likely to identify the question as higher order. Previous work has found the heavy use of pattern recognition by examinees [3], while other studies suggest that higher performing students utilize both clinical reasoning behavior such as pattern recognition and test-taking strategies to rule out alternatives [15].

These results suggest that although faculty intend to write higher- or lower-order MCQs, students’ perception of these questions is more dependent on their knowledge and performance than Bloom’s taxonomy. Previous work has shown that faculty may have difficulty targeting a specific level of taxonomy [10, 11]. We attempted to improve validity of the Bloom’s rating by ensuring that the classification of items by a group of faculty that represented perspectives that were knowledgeable about content as well as those that were less experienced. These perspectives differ from the student perspective in that students may take the shortest way to reach the correct answer so that when the patterns in the question are well known, students do not need to use analysis. This approach may be facilitated by the many commercially available study aids which emphasize pattern recognition. If our ultimate goal with MCQs is to measure clinical reasoning skills required of practicing physicians, we need to better understand how students approach MCQs and how their existing fund of knowledge impacts their approach to MCQs. For instance, only 1.5% of students answered question 8 incorrectly, while 81% labeled it as lower order, despite the faculty labeling it as higher order. We believe this is because they utilized pattern recognition rather than analytic thinking in order to answer this question. This suggests that perhaps, the transition from walking to running (i.e., recall versus analytical thinking) is not as easily identified by Bloom’s taxonomy.

We also find it interesting that students’ performance did not significantly predict their perceptions of question level. One potential explanation for this finding relates to the Dunning-Kruger effect [17]; learners’ self-assessments are unreliable. In this case, a learner with lower performance has an inflated assumption of their ability or knowledge and is less able to accurately identify the items that require higher-order thinking rather than rote recall. Our hypothesis that students with lower performance would perceive more questions as higher order was not confirmed. If students are struggling and have not fully developed analytical thinking skills, they may perceive their low performance as a problem with recall rather than an ability to analyze the question. Further data will be necessary to better explore this question.

Taken together, these results suggest that student understanding of Bloom’s taxonomy, in the way that we currently classify items, may be unrelated to development of clinical reasoning skills. However, faculty should continue to utilize Bloom’s taxonomy in order to write MCQs that test the analytic thinking needed to develop clinical reasoning skills. The transition from walking to running is more than simply knowing more than you did yesterday but also how to apply that knowledge. Helping students develop a deep understanding as well as the ability to apply the knowledge is important in both teaching, learning, and designing assessments. Writing questions that scaffold this process is important step in the process of gaining higher-order reasoning skills and applying the foundational sciences to clinical practice.

We aimed to avoid the challenge of applying a Bloom’s classification to items within the team’s expertise by ensuring that ratings were assigned by consensus. We see it as an opportunity to expand our understanding of higher and lower order. By rating items as a team, we hoped to present a more complete understanding of the definition of higher- and lower-order thinking. As there is no “gold standard” of higher order or lower order, this may be considered a limitation.

We see this study as laying the groundwork for future work to both extend the impact of these findings and to address several key limitations. First, students’ understanding of Bloom’s taxonomy is core to this study, but the concept was only presented briefly prior to students taking the test. Thus, we cannot be certain that emergent differences in these findings are not due to a lack of understanding of the taxonomy on the learners’ parts. For future administrations, students should be provided with the option to respond that they do not know the classification so that they are not forced into responding in an unconfident manner. Similarly, we administered these practice questions immediately following a review session, which may have artificially primed and inflated students’ knowledge at that point in time. To address these concerns, we plan future research using qualitative methods to engage in cognitive interviewing and focus groups to better capture the processes students use as they answer these questions. This will help us better understand the development of clinical reasoning skills, including pattern recognition and higher-order thinking, as students move through the medical curriculum. In addition, expanding the work to other areas of the medical school curriculum may provide additional insight.

In conclusion, this study demonstrated that students did not perceive questions in the same category of Blooms (higher or lower order) compared to the category intended by faculty who wrote the question. Less confident students engaged in higher order, analytic thinking even if the question was intended to be lower order. In addition, students who responded incorrectly to the MCQ were more likely to identify a question as higher order than their peers who responded correctly. These findings demonstrate that students may not approach questions as faculty intend and instead may approach questions based on their confidence, learning approach, and knowledge. This provides additional support to the idea behind our methodological decision to arrive at item classifications via consensus building. These factors that shaped the students’ interactions with the questions also shape the perspectives of those writing or classifying them. Ultimately, it seems as though an item’s classification as higher or lower order is in the eye of the beholder. There is distinct value in observing a learner’s transition towards higher level clinical reasoning, but it may be done best at a level that allows the individual differences between learners to play a greater role than the structures we as educators wish to impose. In this way, moving from walking to running can be more about individual development and concerned faculty should be quick to include the learner as one of the perspectives needed to truly decide if an item is higher or lower order.
